# Prevalence of Reasons for Tooth Extraction in Small- and Medium-Breed Dogs

**DOI:** 10.3390/ani15020224

**Published:** 2025-01-15

**Authors:** Chun-Geun Kim, Daehyun Kwon, Kyuyoung Lee, Se Eun Kim, Hyun Min Jo

**Affiliations:** 1 Evichi Veterinary Dental Hospital, Seoul 06062, Republic of Korea; vetgold@hanmail.net; 2 May Veterinary Dental Hospital, Seoul 06240, Republic of Korea; vetcafe@hanmail.net; 3 Department of Veterinary Surgery, College of Veterinary Medicine and BK21 Plus Project Team, Chonnam National University, Gwangju 61186, Republic of Korea; 4 Department of Microbiology, Institute for Viral Diseases, College of Medicine, Korea University, Seoul 02841, Republic of Korea; wing290@korea.ac.kr; 5 Biomaterial R&BD Center, Chonnam National University, Gwangju 61186, Republic of Korea

**Keywords:** companion animals, dental diseases, periodontal disease, small-breed dogs, tooth extraction

## Abstract

As the life span of companion animals increases and their owners’ concern for their quality of life increases, interest in dental diseases has also gained attention. While many studies have explored oral diseases in general, research on small-breed dogs remains limited. Therefore, in this study, we analyzed various diseases that led to tooth extraction in small- and medium-breed dogs. A study involving 2201 client-owned small- and medium-breed dogs was conducted. All dogs were categorized by sex, age, breed, and tooth position. Teeth were classified into untreated, missing, or extracted categories. For extracted teeth, 14 causes were identified. The results revealed that periodontal disease was the primary cause of tooth extractions, particularly in older dogs and small breeds. In addition to periodontal disease, various other diseases that led to tooth extraction were identified. For these other diseases, the number of extracted teeth increased with age, and brachycephalic breeds exhibited higher extraction rates. The study emphasizes the need for personalized oral care and further research into other contributing factors such as diet and genetics to better understand and manage oral health in dogs. It also offers valuable insights into the broader patterns of tooth extraction associated with various dental diseases.

## Simple Summary

As companion animals live longer and their owners become more concerned with their quality of life, dental health in dogs is gaining attention. Dental diseases are common in dogs, especially small breeds, but research on this group is limited. This study analyzed tooth extractions in 2201 small- and medium-breed dogs to understand the main causes and patterns. Older dogs and small breeds were the most affected by periodontal disease, which was found to be the leading cause of extractions. Other factors, such as tooth fractures and certain oral diseases, also led to tooth extractions. This research highlights the importance of personalized dental care for dogs, particularly as they age, and suggests the need for further studies on how diet, genetics, and other factors affect their oral health. By improving our understanding of these issues, we can enhance the overall wellbeing of our canine companions.

## 1. Introduction

As companion animals have longer lifespans and their owners become more concerned about their pets’ quality of life, interest in veterinary dentistry is growing. In the case of oral disease, timely prophylaxis and treatment are crucial to prevent oral abnormalities including pain, loss of appetite, drooling, bad breath, and the potential development of systemic or local abnormalities [1,2]. Therefore, accurate diagnosis and appropriate treatment of oral problems have a significant impact on the overall health and wellbeing of the animal. The gold standard for veterinary oral health care includes oral examination, radiography, dental prophylaxis, and treatment [3].

The oral cavity of dogs is susceptible to a wide variety of diseases [4]. Epidemiological studies have consistently identified periodontal disease and dental calculus as the most prevalent oral conditions in dogs [4,5]. Other significant conditions include missing teeth, abnormal attrition, dental caries, and tumors. When oral abnormalities arise, various treatments are available depending on the causative disease, such as tooth extraction, endodontic treatment, periodontal treatment, restorations, and orthodontics. Despite the availability of these treatments, tooth extraction remains the most commonly used method in veterinary dentistry [6,7]. Factors necessitating tooth extraction include periodontal disease, tooth resorption, tooth fractures, endodontic and periapical diseases, caries, malocclusion, and retained roots.

According to the research findings of Elseddawy and Whyte, periodontal disease is highly prevalent in dogs and often requires tooth extraction [5,8]. Periodontal disease encompasses gingivitis, which involves inflammation of the soft tissues, and periodontitis, which is characterized by the destruction of both bone and soft tissues. It is classified into various stages based on clinical symptoms [6], which are diverse and include halitosis, drooling, and anorexia. As the disease progresses, vertical or horizontal bone loss in the alveolar bone may occur, leading to increased tooth mobility. In cases of mild periodontal disease, treatments such as scaling, root planing, and antibiotic therapy are typically employed [9]. However, significant clinical tooth mobility or attachment loss of 50% or more generally indicates the need for surgical extraction.

While numerous studies have explored the prevalence of dental diseases to develop precise treatment plans and facilitate client consultations, there is limited research focusing specifically on the prevalence of causes for tooth extraction [10,11,12,13]. Understanding the causes of tooth extraction based on various criteria is essential for developing targeted preventive measures and promoting optimal oral health. In addition, it could establish a robust standard for selecting tooth extraction over other dental surgery methods in the future. Many existing studies predominantly focus on medium- and large-breed dogs [10,11,13]. However, small-breed dogs, with their distinctive jaw structures and dental anatomy, such as crowded teeth and narrow dental arches, may experience unique challenges that make them more susceptible to certain dental conditions. Moreover, breed-specific predispositions to dental diseases could influence the prevalence and causes of tooth extractions in these dogs.

Therefore, this retrospective study aims to analyze the prevalence of reasons for tooth extraction in small- and medium-breed dogs by considering factors such as sex, age, breed, and tooth position. By identifying the most common dental diseases leading to extraction in these breeds, we seek to contribute to the development of breed-specific preventive and therapeutic strategies. The findings are also intended to raise awareness among practitioners about the primary oral issues faced by these dogs and to facilitate effective communication with clients and pet owners regarding these significant disorders. Ultimately, the purpose of this study was to classify the causes of tooth extraction through a retrospective analysis small- and medium-breed dogs and to promote oral health through the prevention and treatment of oral diseases based on the identified causes.

## 2. Materials and Methods

### 2.1. Case Inclusion

A total of 2201 client-owned small- and medium-breed dogs included were presented for dental assessment and treatment between January 2015 and June 2021. Of these, 1271 dogs were presented to Evichi Veterinary Dental Hospital (EVDH, C.-G.K.), and 930 were presented to May Veterinary Dental Hospital (MVDH, D.-H.K.). Dogs presented for professional teeth cleaning and extraction of persistent deciduous teeth were excluded from this study, and only dogs that had at least one permanent tooth extracted were included. Dental and oral surgeries other than tooth extractions were beyond the scope of this study. Additionally, previously extracted teeth were classified as missing. The timing of extractions was recorded only during a single visit to the hospital, and cases in which the same dog underwent additional extractions during the study period were excluded.

### 2.2. Medical Record Review

Each dog’s information was obtained from their electronic medical records including their history, physical examination findings, laboratory diagnostics, diagnostic imaging results, oral pictures, and dental charts.

### 2.3. Intraoral Radiography

All dogs included in this study underwent a full-mouth radiographic examination using a standardized system of positioning, views, and techniques [3,14]. The radiographs were obtained using a standard, wall-mounted dental radiography unit (Progency^®^ Dental, Midmark, Seoul, Republic of Korea) with a direct digital imaging system with a No. 2 sensor (EVA-VET; AFP Imaging, Elmsford, NY, USA) and photostimulable phosphor plate (PSP) system (CR7VET, im3, SHINKI, Seoul, Republic of Korea) with size No. 0 to 4 plates. The radiographic interpretation was performed by each veterinarian (C.-G.K. and D.-H.K.).

### 2.4. Cone-Beam Computed Tomography (CBCT)

Some individuals underwent CBCT to accurately evaluate factors that could be overlooked by X-rays or oral examinations when determining which teeth required extraction. Preoperative CBCT (NewTom 5GXL VET CBCT scanner, NewTom, Verona, Italy) scans were obtained to assess the anatomical and structural features of the skull and dentition. The field of view and voxel size varied depending on the size of each dog’s skull. The images were evaluated using Invivo5 software (Anatomage, San Jose, CA, USA). The CBCT scans were performed only at EVDH from September 2017 until the end of the study.

### 2.5. Thorough Oral Examination and Dental Chart Recording

Systematic oral examinations were performed by C.-G.K. and D.-H.K. under the same conditions, including various indices and classifications [15,16,17], and the results were recorded by well-trained technicians.

### 2.6. Tooth Extraction Methods

Extraction was performed using the recommended instruments and equipment and surgical techniques [6,18,19,20,21]. Simple extraction, surgical extractions, and coronectomy (crown amputation with intentional root retention) were performed selectively for single- or multi-rooted teeth and depending on the pathology of the tooth and alveolar bone. The principle for tooth extraction was to remove all tooth roots. Additionally, regardless of whether tooth sectioning was performed based on the number of roots, each extracted tooth, whether single-rooted or multi-rooted, was counted as one. All extraction sites were debrided of inflamed tissue, and flaps were elevated and sutured without tension.

### 2.7. Classification of Causes for Extractions

The study categorized dogs by sex, age, breed, and tooth position. A total of 2201 dogs with 92,442 teeth were classified into three distinct categories based on their condition: untreated teeth, missing teeth, and extracted teeth. Furthermore, within the subset of extracted teeth, the causes of tooth extraction were further categorized into 14 distinct categories for detailed analysis. The following table presents the names of these categories of disease that led to tooth extractions and their corresponding abbreviations (Table 1).

### 2.8. Statistical Analysis

Descriptive statistics were used to evaluate the distribution of oral diseases leading to tooth extraction in dogs, utilizing frequency tables and graphs based on data from electronic medical records. We performed one-way analysis of variance (ANOVA) to evaluate the association of the number of tooth extractions or periodontal diseases with demographic factors (α = 0.05). Tukey’s honestly significant difference (HSD) test was conducted to compare the mean number of tooth extractions or periodontal diseases in each pair of groups of a demographic factor as the post hoc test of one-way ANOVA.

## 3. Results

### 3.1. Distribution of Dogs and Extracted Teeth in 2201 Small- and Medium-Breed Dogs According to Various Classifications

#### 3.1.1. Distribution of Dogs by Sex, Age, and Breed

**Figure 1:**
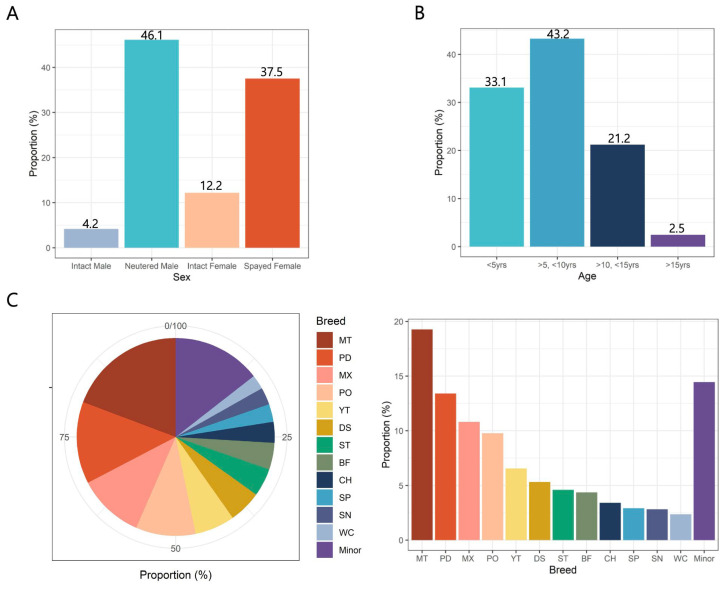
Distribution of tooth extraction in 2201 small- and medium-breed dogs according to various classifications. (**A**) Distribution of sex. (**B**) Distribution of age. (**C**) Distribution of breed. MT, Maltese; PD, Miniature Poodle; MX, Mixed; PO, Pomeranian; YT, Yorkshire Terrier; DS, Dachshund; ST, Shih Tzu; BF, Bichon Frise; CH, Chihuahua; SP, Spitz; SN, Miniature Schnauzer; WC, Welsh Corgi.

We conducted a comprehensive analysis of 2201 dogs of 31 breeds to investigate factors contributing to tooth extraction and focused on the influence of sex, age, and breed. With regard to sex, 1015 (46.1%) were neutered males, 826 (37.5%) were spayed females, 268 (12.2%) were intact females, and 92 (4.2%) were intact males. Notably, the number of spayed/neutered dogs (83.6%) far exceeded intact dogs (16.4%) (Figure 1A). When categorized by age, Group 2 (dogs aged between 6 and 10 years) had the highest representation with 952 dogs (43.2%), followed by Group 1 (under 5 years) with 728 dogs (33.1%), Group 3 (11 to 15 years) with 467 dogs (21.2%), and Group 4 (over 16 years) with 54 dogs (2.5%). Most dogs in the study were younger than 10 years old (76.3%) (Figure 1B). For breed classification, we defined major breeds as those with more than 50 dogs. The major breeds included Maltese, Miniature Poodle, mixed breeds, Pomeranian, Yorkshire Terrier, Dachshund, Shih Tzu, Bichon Frise, Chihuahua, Spitz, Schnauzer, and Welsh Corgi, which are predominantly small dog breeds (Figure 1C). Breeds with fewer than 50 dogs included were classified as minor breeds.

#### 3.1.2. Distribution of All Teeth in 2201 Small- and Medium-Breed Dogs

**Figure 2:**
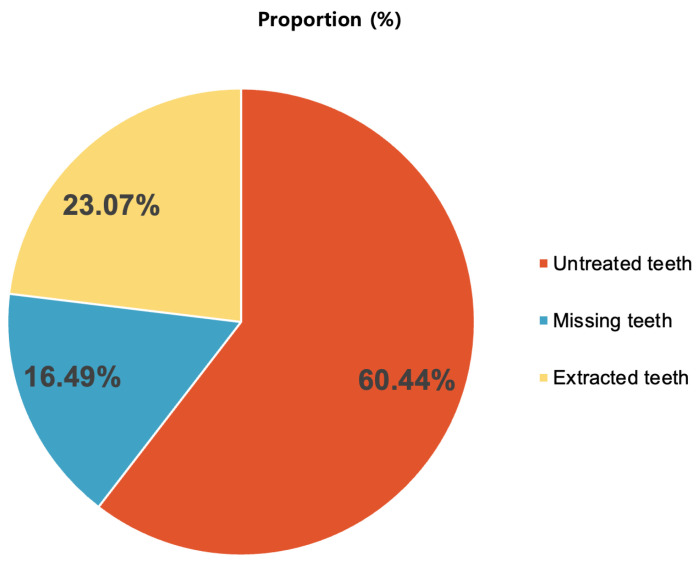
Proportions of untreated, missing, and extracted teeth from a total of 92,442 teeth in 2201 small- and medium-breed dogs.

A comprehensive analysis of all teeth included in the study revealed that among the 92,442 teeth assessed, 60.44% did not require extraction, 16.49% were missing, and 23.07% underwent tooth extraction (Figure 2).

#### 3.1.3. Distribution of Tooth Extractions Due to Various Oral Diseases

**Figure 3:**
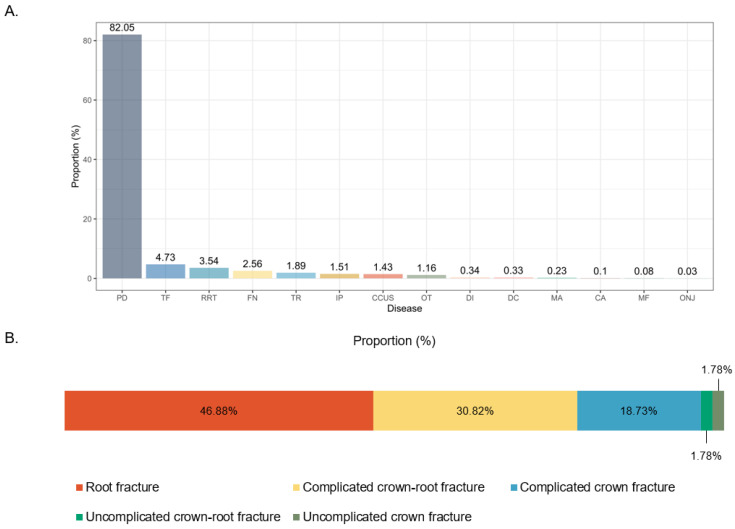
Summary of the distribution of tooth extraction rates due to various causes. (**A**) Proportion of causes of tooth extraction by each oral disease. (**B**) Distribution of fractured teeth due to various oral diseases. PD: Periodontal disease; TF: Tooth fracture; RRT: Retained root tip; FN: Failure to narrow of the pulp cavity; TR: Tooth resorption; IP: Impacted teeth; CCUS: Canine chronic ulcerative stomatitis; OT: Oral tumor-engulfed teeth; DI: Dens invaginatus; DC: Dentigerous cyst; MA: Malocclusion; CA: Caries; MF: Mandibular fracture; ONJ: Osteonecrosis of the jaw.

A subsequent analysis was performed to evaluate the various causes of tooth extraction. Tooth extraction due to periodontal disease accounted for an overwhelmingly high proportion of 82.05%, followed by tooth fractures (4.73%), retained root tips (3.54%), failure to narrow of the pulp cavity (2.56%), tooth resorption (1.89%), impacted teeth (1.51%), canine chronic ulcerative stomatitis (CCUS) (1.43%), and oral tumor-engulfed teeth (1.16%) (Figure 3A). Excluding teeth extracted due to periodontal disease, extracted teeth were categorized based on their proportions: conditions accounting for more than 1.0% of extractions were considered major causes, while those accounting for less than 1.0% of extractions were classified as minor causes. The major causes for tooth extraction included tooth fractures, retained root tips, failure to narrow of the pulp cavity, tooth resorption, impacted teeth, CCUS, and oral tumor-engulfed teeth. On the other hand, the minor causes of tooth extraction included dens invaginatus, dentigerous cysts, malocclusion, caries (tooth decay), mandibular fractures, and osteonecrosis. In addition, in our research, tooth fractures were categorized into five types: complicated crown fractures, complicated crown-root fractures, root fractures, uncomplicated crown fractures, and uncomplicated crown-root fractures. Among tooth extractions due to fractures, root fractures accounted for the highest proportion (46.88%), followed by complicated crown-root fractures (30.82%), complicated crown fractures (18.73%), uncomplicated crown-root fractures (1.78%), and uncomplicated crown fractures (1.78%) (Figure 3B).

#### 3.1.4. Prevalence of Oral Disease in Each Tooth Among the Maxillary and Mandibular Extracted Teeth in 2201 Small- and Medium-Breed Dogs

A detailed analysis of each tooth showed periodontal disease as the major reason for extraction in all extracted teeth. In addition, the maxillary fourth premolar and mandibular first molar teeth exhibited high rates of complicated crown-root fracture. Furthermore, impacted teeth were observed at significantly higher rates in the mandibular first premolars than in other teeth (Table 2 and Table 3).

### 3.2. Distribution of Extracted Teeth Due to Periodontal Disease in 2201 Small- and Medium-Breed Dogs

#### 3.2.1. Distribution of Extracted Teeth Due to Periodontal Disease by Sex, Age, and Breed

**Figure 4:**
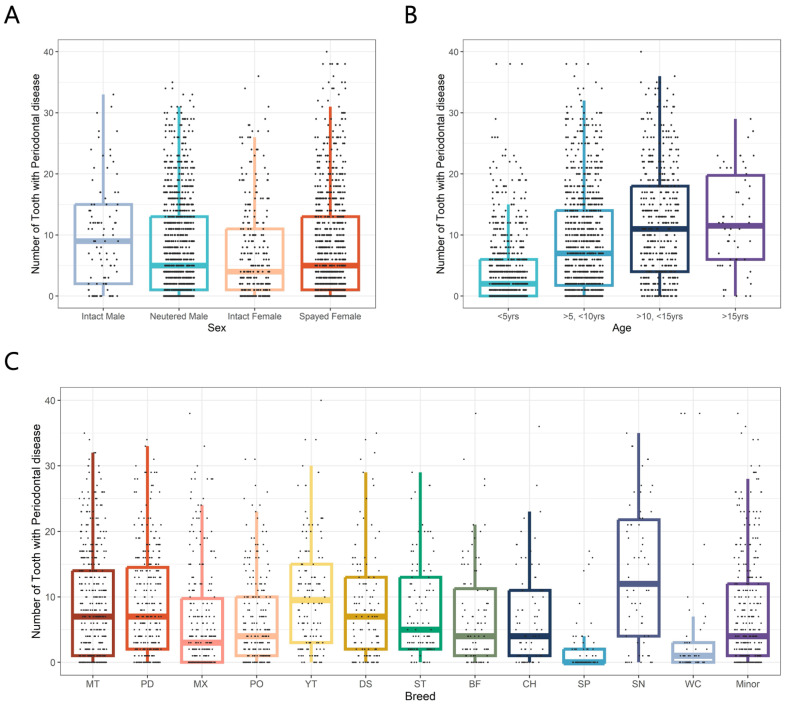
Distribution of extracted teeth due to periodontal disease in 2021 small- and medium-breed dogs according to various classifications. (**A**) Distribution of sex. (**B**) Distribution of age. (**C**) Distribution of breed. The point presents observations of our study, and the color-coded box plot displays the distribution of each observation, including the median as well as the 25th and 75th percentiles.

As mentioned above, among various causes of tooth extraction, periodontal disease was the most common, prompting a focused statistical analysis. Sex-based comparisons revealed that intact males had the highest average number of extracted teeth due to periodontal disease (9.93), followed by spayed females (8.19), neutered males (7.78), and intact females (7.15). Overall, there was no statistically significant difference observed across the sexes, except for a notable distinction between intact males and intact females (*p* < 0.05) (Figure 4A).

Age-based analysis showed that the average number of extracted teeth due to periodontal disease increased with age. Group 1 (under 5 years) had an average of 6.50 extracted teeth, Group 2 (dogs aged between 6 and 10 years) had 8.65, Group 3 (11 to 15 years) had 12.1, and Group 4 (over 16 years) had 12.2. Statistical analysis confirmed a significant increasing trend in the number of extracted teeth due to periodontal disease with advancing age (*p* < 0.01) (Figure 4B).

Breed comparisons revealed significant differences in the average number of extracted teeth due to periodontal disease (*p* < 0.05). Schnauzers had the highest average number of extracted teeth (13.7), followed by Yorkshire Terriers (10.4) and Miniature Poodles (8.98). In contrast, mixed breeds, Welsh Corgis, and Spitz exhibited less extracted teeth, with averages of 6.48, 4.25, and 2.03 affected teeth, respectively. While there were differences in number of extracted teeth due to periodontal disease among breeds, no significant differences were observed based on breed size (Figure 4C).

#### 3.2.2. Distribution of Extracted Teeth Due to Periodontal Disease by Tooth Position

**Figure 5:**
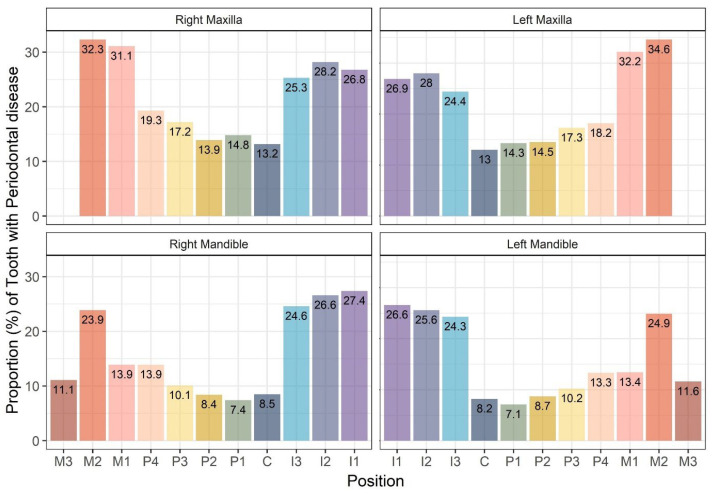
Distribution of extracted teeth due to periodontal disease in small- and medium-breed dogs according to tooth position. C: canine; I: incisor; M: molar; P: premolar teeth.

Analyzing tooth positions within the total of 42 teeth, we found that in the maxilla (upper jaw), the left and right second molar teeth had the highest extraction rates due to periodontal disease, with rates of 34.6% and 32.3%, respectively. This was followed by the left and right first molar teeth, with rates of 32.2% and 31.1%, respectively. In the mandible (lower jaw), the left and right first incisor teeth exhibited the highest extraction rates at 26.6% and 27.4%, respectively, followed by the left and right second incisor teeth at 25.6% and 26.6%, respectively. In both jaws, canine and premolar teeth showed relatively low extraction rates due to periodontal disease (Figure 5).

### 3.3. Distribution of Extracted Teeth Due to Causes Other than Periodontal Disease in 2021 Small- and Medium-Breed Dogs

#### 3.3.1. Distribution of Extracted Teeth Excluding Periodontal Disease by Sex, Age, and Breed

**Figure 6:**
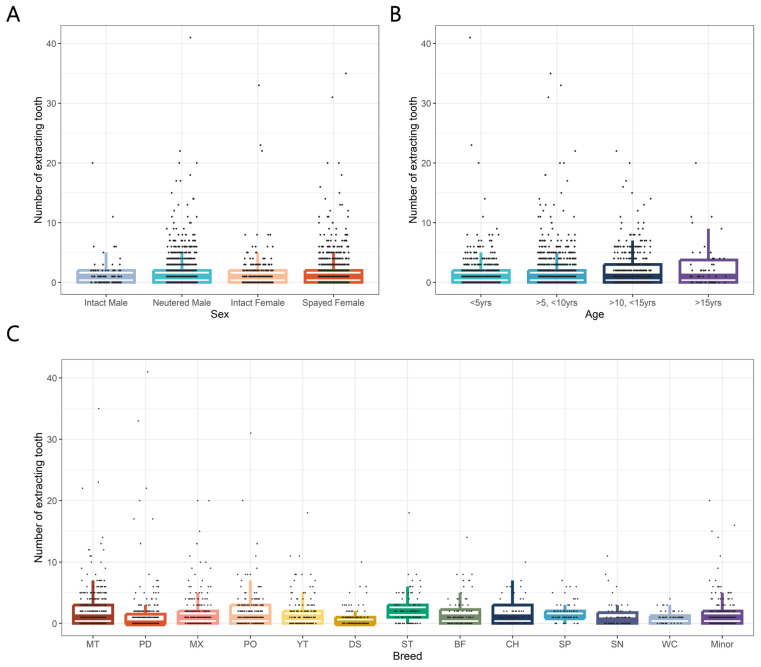
Distribution of extracted teeth due to causes other than periodontal disease in 2021 small- and medium-breed dogs according to various classifications. (**A**) Distribution of sex. (**B**) Distribution of age. (**C**) Distribution of breed. The point presents observations of our study, and the color-coded box plot displays the distribution of each observation, including the median as well as the 25th and 75th percentiles.

We conducted a statistical analysis of the various causes of tooth extraction excluding periodontal disease. In the sex-based comparison, the average number of extracted teeth due to other causes was similar across sexes: intact males (1.35), neutered males (1.81), intact females (1.46), and spayed females (1.81). No statistically significant differences were observed among the sex groups. Notably, the number of extracted teeth due to other diseases was significantly lower than those extracted due to periodontal disease across all sex groups (Figure 6A).

Age-based analysis revealed that the average number of extracted teeth increased with age: Group 1 (under 5 years) had 1.43 extractions, Group 2 (dogs aged between 6 and 10 years) had 1.79, Group 3 (11 to 15 years) had 2.07, and Group 4 (over 16 years) had 2.56. This indicates an increasing trend in the number of extracted teeth due to causes other than periodontal disease as age advanced. In particular, Groups 3 and 4 exhibited statistically significant differences compared with Group 1 (Group 3 vs. Group 1, *p* < 0.01; Group 4 vs. Group 1, *p* < 0.05) (Figure 6B).

In terms of breed comparison, Shih Tzus had the highest average numbers of extracted teeth (2.25), followed by Maltese (2.16) and Miniature Poodles (2.01). In contrast, Schnauzers, Welsh Corgis, and Dachshunds demonstrated lower average numbers of extracted teeth, with values of 1.27, 1.10, and 0.94, respectively. No significant differences were found among breeds (Figure 6C).

#### 3.3.2. Distribution of Extracted Teeth Due to Causes Other than Periodontal Disease by Sex, Age, and Breed

**Figure 7:**
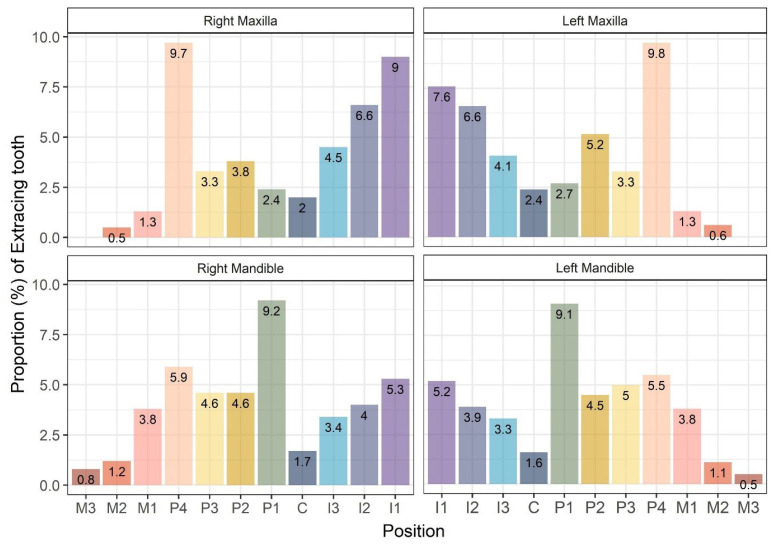
Distribution of extracted teeth due to causes other than periodontal disease in small- and medium-breed dogs according to the position of the teeth.

When comparing tooth positions, in the upper jaw, the left and right fourth premolar teeth had the highest extraction rates of 9.8% and 9.7%, respectively. This was followed by the left and right first incisor teeth, with rates of 7.6% and 9.0%, respectively. In the lower jaw, the left and right first premolar teeth had higher extraction rates of 9.1% and 9.2%, respectively, followed by the left and right fourth premolar teeth at 5.5% and 5.9%, respectively. Canine and the second and third molar teeth had relatively low extraction rates in both jaws (Figure 7).

## 4. Discussion

This study analyzed various oral disorders that lead to tooth extraction in dogs, and periodontal disease was identified as the primary cause. To provide a more comprehensive assessment, we categorized the cases into those associated with periodontal disease alone and those involving other oral diseases.

When classifying the cases, it became evident that spayed/neutered animals were significantly more prevalent than intact ones. This may be attributed to the prevalent practice of spaying and neutering, as observed in another research study [22,23]. Examining the age distribution, most dogs were under 10 years old, and fewer cases involved dogs aged 15 years or older. As for the breed distribution, the high representation of small breeds, which aligns with the predominance of small dog ownership in urbanized environments, further emphasizes the breed-related predisposition to oral health issues.

Periodontal disease, which includes conditions such as gingivitis (soft tissue inflammation) and periodontitis (bone and tissue destruction), is typically categorized into stages based on clinical symptoms [24]. As periodontal disease progresses, clinical signs such as halitosis, drooling, and anorexia become more prominent, and observable bone loss occurs in the alveolar regions, leading to increased tooth mobility [7]. Mild periodontal disease can often be managed with non-surgical treatments including scaling, root planing, and antibiotic therapy [9]. However, in cases where there is substantial tooth mobility or more than 50% attachment loss, surgical extraction becomes necessary [6].

The factors contributing to periodontal disease are multifaceted and complex. Our sex-based analysis revealed that intact males had the highest rate of tooth extractions, while intact females had the lowest. Neutered males and spayed females exhibited relatively similar extraction rates. Alveolar bone loss is known to serve as a significant criterion when deciding whether tooth extraction is necessary for periodontal disease cases. Some studies have provided evidence that the female hormone estrogen can slow alveolar bone loss [25]. Delaying this type of bone loss may reduce the incidence of tooth extraction due to the progression of periodontal disease in females compared with males. Therefore, the disparity in tooth extraction rates between intact males and females may be partially attributed to hormonal differences.

Age was another significant factor, as this study found that older dogs had significantly more teeth extracted due to periodontal disease. This finding aligns with studies by Wallis and Harvey, which demonstrated an increased prevalence of periodontal disease with age [10,13]. The progressive nature of periodontal disease is that without treatment, symptoms worsen over time, leading to increased tooth mobility and attachment loss [24,26]. Older dogs also tend to develop more uneven tooth surfaces, produce less saliva, and have reduced chewing activity, which makes it easier for plaque and tartar to form and accumulate [27]; their weakened immune systems also make them more susceptible to gingival and periodontal inflammation [28]. As a result, the likelihood of developing periodontal disease increases with age, leading to an increased rate of tooth extraction. This suggests a linear correlation between age and the number of tooth extractions. These findings highlight the importance of early treatment and routine oral care to prevent periodontal disease progression, especially in older dogs.

In terms of breed differences, more tooth extractions were observed in breeds such as Miniature Poodles, Miniature Schnauzers, and Yorkshire Terriers, which are categorized as small-breed dogs. These breeds have been identified in previous studies as breeds with a relatively high prevalence of periodontal disease [10]. In contrast, relatively fewer extractions were found in mixed breeds, Welsh Corgis, and Spitzes, which can be classified as small- to medium-breed dogs. Previous studies have shown that extra small- to small-breed dogs have a larger ratio of tooth size to skull size than medium- to large-breed dogs [10]. This tooth-to-skull size discrepancy can lead to teeth crowding, which in turn increases the likelihood of calculus or plaque deposition between the teeth, thus increasing the risk of periodontal disease [10]. It is important to note that this study lacked data on large-breed dogs. Nonetheless, consistent with the findings of previous studies. A comparison with prior studies reveals strong consistencies. Wallis et al. also found that periodontal disease predominantly affects small-breed dogs, which aligns with our observations regarding the high susceptibility of breeds like Miniature Schnauzers and Yorkshire Terriers [10]. However, no significant differences in extraction rates were observed among breeds of similar sizes, suggesting that the body size of dogs plays a more crucial role than breed specificity in this context [29] 

In addition, when evaluating tooth extraction rates due to periodontal disease according to tooth position, the first and second molar teeth had the highest extraction rates in the maxilla (upper jaw), followed by the incisor teeth. In the mandible (lower jaw), the highest extraction rates occurred in the incisor and second molar teeth. The extraction rates in the canine and premolar teeth were relatively low. Our findings showed higher extraction rates for incisor teeth, which is inconsistent with studies on the degree of calculus deposition by tooth position. Previous research comparing major teeth (canine, second premolar, third premolar, fourth premolar, and first molar) found a high calculus index in the fourth premolar and first molar teeth. However, in the case of the fourth premolar teeth, the occurrence of tooth mobility, which is one of the criteria for determining extraction, was relatively low compared with other teeth. On the other hand, for incisor teeth, although the calculus index was not accurately measured, the mobility of these teeth was higher than that of other teeth [13]. Excessive calculus deposition is generally considered the direct cause of periodontal disease. However, the appropriate treatment for periodontal disease can be divided into conservative treatments and tooth extraction depending on the degree of tooth mobility and attachment loss. Incisor teeth have shorter roots and a more conical shape, which makes them more susceptible to tooth mobility as a result of periodontal tissue loss. The relatively shallow root depth of the incisors makes them particularly vulnerable to the destructive effects of periodontal disease, contributing to a higher incidence of extractions in these cases.

Second, an analysis was conducted on cases of tooth extraction due to diseases other than periodontal disease. Tooth fractures were most frequently observed in the maxillary fourth premolar and mandibular first molar teeth. In dogs, these teeth, also known as the carnassial teeth, are specialized for incising and crushing hard materials such as large pieces of meat or bones [30]. Consequently, fractures are more likely to occur during the chewing of hard foods and materials in the maxillary fourth premolar and mandibular first molar teeth than in other teeth. When examining the diseases related to tooth fractures, the extent of tooth extraction varied depending on whether the pulp was exposed and whether the root was fractured. For cases of tooth fracture, there is an additional treatment option known as endodontic treatment, which is an alternative to the standard tooth extraction. The presence of pulp infection is a critical factor in determining the appropriate treatment for fractured teeth [31]. In cases of complicated crown-root fractures or complicated crown fractures, pulp exposure typically occurs. Treatment of tooth fractures with pulp exposure and periapical lesions can be treated with a root canal treatment as one of the treatment options. Root canal treatment is less invasive than extraction but requires at least one follow-up dental X-ray examination under general anesthesia to monitor the progress of the treatment. If the patient is not suitable for repeated anesthesia or the financial situation of the dog’s owner does not allow this procedure, extraction can be considered. Patients often visit the hospital after a significant period following the fracture, by which time a pulp infection has usually developed. Consequently, as observed in the results of this study, extraction is frequently the chosen treatment. However, in general, uncomplicated fractures in which the pulp is not exposed are treated with bonded sealants instead of resorting to tooth extraction [32]. Therefore, from the available data, it is evident that the frequency of extractions for uncomplicated crown fractures and uncomplicated crown-root fractures is quite low.

Root fractures and retained roots can arise from a variety of factors including trauma, incomplete tooth extraction, and advanced tooth resorption. This condition occurs when the visible crown of the tooth is lost, leaving only the root embedded in the alveolar bone [33]. Neglecting to extract the retained root can lead to inflammation, infection, osteomyelitis, gum inflammation, and persistent pain, making extraction the recommended course of action [34]. This is one of the most common complications of oral surgery. Our data show a relatively high frequency of tooth extractions due to retained roots and root fractures.

Failure to narrow of the pulp cavity is a disease that signifies an abnormality in the dental pulp structure. Ordinarily, as age advances, secondary dentin is consistently generated by ameloblasts and odontoblasts, leading to a narrowing of the pulp chamber [35]. However, when ameloblasts and odontoblasts are damaged due to causes such as tooth trauma, the size of the root canal does not narrow and it becomes stagnant [36,37]. In particular, the occurrence of such trauma is high due to the provision of various chewing materials, such as hard dental treats for tartar removal. This factor contributed to the elevated tooth extraction rate observed in our data.

According to our study, the highest incidence of impacted teeth was observed in the mandibular first premolar teeth, and the occurrence of dentigerous cysts was also observed to be higher in these teeth than in other teeth. In addition, teeth in the same location on the left and right sides showed similar prevalences overall. In the case of impacted teeth, these teeth remain unable to emerge and remain buried within the alveolar bone; this condition is often seen in small-breed dogs with compact skulls [38]. These impacted teeth are typically not readily visible without the aid of dental X-rays. Failing to address an impacted tooth can lead to the development of dentigerous cysts, making extractions the recommended treatment [38]. Our data demonstrate a relatively high incidence of tooth extractions due to this condition. The mandibular first premolar teeth are among the most commonly missing teeth in dogs [39]. However, the findings of this study indicate that unerupted teeth may be present even when not visible during clinical examination, and if unerupted teeth are found, intraoral radiographic examination is essential for an accurate evaluation.

CCUS is a common disease in dogs that can occur in all breeds but is particularly prevalent in white, small-breed dogs (especially Maltese) [40,41]. Treatments such as plaque removal through scaling or home care can be applied depending on the severity of the disease. However, due to its frequent association with periodontal disease, a significant number of dogs may require tooth extractions to manage the condition effectively [40,42]. Our study results showed that CCUS was the sixth most common cause of tooth extraction, excluding periodontal disease, and this is thought to be due to Maltese being the most prevalent breed in the entire sample.

Next, there were tumor-engulfed and oral tumor biopsy-related extractions. Oral tumors are common in dogs, especially older dogs. If a tumor is detected, surgical removal is performed depending on the type and size of the tumor, or if the tumor is very large, maxillectomy or mandibulectomy is performed, and in this process, nearby teeth are extracted [43]. In dogs, oral tumors can be classified into odontogenic and non-odontogenic tumors. Common malignant oral tumors observed in dogs include malignant melanoma, squamous cell carcinoma, and fibrosarcoma, which are non-odontogenic tumors [44]. These tumors require more extensive oral surgery rather than a simple tooth extraction. Non-odontogenic tumor removal surgery may also involve simultaneous removal of teeth. As a result, tooth extractions in these cases are not considered the primary treatment for tumors but are rather one of the procedures involved in oral surgery. Therefore, it is understandable that the number of extractions related to oral tumors appears relatively low in the available data, despite the common occurrence of oral tumors.

In terms of minor diseases, malocclusion is a relatively common dental abnormality in veterinary medicine. There are various treatment options depending on the type or degree of malocclusion, and although extraction is a viable treatment option [45,46], this condition can also be treated with orthodontics; thus, the distribution of extractions was low. Finally, conditions such as dens invaginatus, caries, and other uncommon diseases are infrequently observed in dogs, resulting in a low occurrence of extractions.

In the subsequent analysis, as a result of comparing and analyzing the degree of tooth extraction caused by the abovementioned diseases by sex, no difference in prevalence was found.

In the analysis by age, it was confirmed that the number of teeth extracted increases with age, as was the case with periodontal disease. This seems to be due to the characteristics of older dogs being more susceptible to diseases such as tumors, which are age-related diseases [47], in addition to periodontal disease and due to it being more difficult to apply conservative treatment in older dogs than in younger dogs.

In the analysis by breed, as with periodontal disease, the number of teeth extracted was high in small breeds such as Shih Tzus, Maltese, and Miniature Poodles. In particular, in dogs with relatively short heads, the number of teeth extracted was high, while in the case of dogs with relatively long heads, such as Welsh Corgis and Dachshunds, the number of teeth extracted was low. This is consistent with the findings of previous studies that have shown that dogs with smaller and shorter heads are more prone to dental disease because their oral cavity area is narrower; therefore, the teeth become crowded [10].

In addition, when evaluating the rates of tooth extraction due to other diseases by tooth position, it was observed that the fourth premolar teeth had the highest rates of extractions in the maxilla, followed by the incisor teeth. In the mandible, the highest rates of extraction occurred in the first premolar teeth. The rates of extraction for canine and molar teeth were relatively low. There was no difference between the left and right sides. In the maxilla, the fourth premolar teeth were more prone to complicated crown-root fractures, while in the mandible, the first premolar teeth were more likely to develop impacted teeth and dentigerous cysts. As discussed, the prevalence of oral diseases other than periodontal disease was relatively low in this study, and, in most cases, no significant variations were identified based on tooth location. This suggests that certain teeth, particularly the maxillary fourth premolar teeth and mandibular first premolar teeth, exhibited relatively higher rates of tooth extraction.

In summary, this study identified periodontal disease as the primary cause of tooth extractions in dogs, with older dogs and small-breed dogs, particularly those with compact skulls, being more susceptible due to issues such as tooth crowding and increased vulnerability to dental problems. Other common causes include tooth fractures, retained root tips, pulp cavity failure to narrow of the pulp cavity, tooth resorption, impacted teeth, CCUS, and oral tumors. Age significantly influences extraction rates, with older dogs having more extractions, while sex differences are minimal; however, intact males showed slightly higher rates linked to periodontal disease. Although small breeds had higher extraction averages, breed species showed limited influence. There are two limitations in this study. First, the number of individuals in each group varied significantly, with a huge disparity between intact males and neutered males. Thus, it is difficult to conclude that sex directly influences tooth extraction due to periodontal disease. Therefore, additional research based on a similar number of individuals is required in the future. Second, most individuals analyzed in this study were small- and medium-breed. Consequently, the distribution of extracted teeth due to variable oral disease in large breeds could not be thoroughly examined due to the limited number of dogs. Therefore, future studies focusing on medium-to-large breeds are necessary to provide comprehensive insights into oral disease that causes tooth extraction across all breed sizes.

## 5. Conclusions

This study analyzed tooth extraction in small- and medium-breed dogs and confirmed that periodontal disease and various other oral diseases are key contributors. Periodontal disease emerged as the leading contributor and was influenced by factors such as sex, age, breed, and tooth position. In contrast, the prevalence of tooth extractions due to other oral diseases showed minimal variation across these factors. Instead, extractions due to other oral diseases were primarily linked to diseases that are both common in dogs and best treated through extraction, and individual environmental factors played a significant role. Based on the results of this retrospective study, the majority of tooth extractions in small- and medium-breed dogs were attributed to periodontal disease. Therefore, implementing preventive measures, such as daily tooth brushing, regular dental examinations, and professional teeth cleaning, and ensuring early diagnosis and treatment are crucial strategies to reduce the incidence of tooth extractions caused by periodontal disease.

## Figures and Tables

**Table 1:** Abbreviations for various oral diseases.

No	Abbreviations	Full Text
1	CA	Caries
2	CCUS	Canine chronic ulcerative stomatitis
3	DI	Dens invaginatus
4	DC	Dentigerous cyst
5	FN	Failure to narrow of the pulp cavity
6	IP	Impacted teeth
7	MA	Malocclusion
8	MF	Mandibular fracture
9	ONJ	Osteonecrosis of the jaw
10	OT	Oral tumor-engulfed teeth
11	PD	Periodontal disease
12	RRT	Retained root tip
13	TF	Tooth fracture
14	TR	Tooth resorption

**Table 2:** Prevalence of oral diseases in each tooth among maxillary extracted teeth.

Prevalence of Oral Disease (%)																				
ONJ	MF	CA	MA	DC	DI	OT	CCUS	IP	TR	TF					FN	RRT	PD	Number of Extracted Teeth	Categories	
										UCF	UCRF	CCF	CCRF	RF						
0.0	0.0	0.1	0.0	0.0	0.0	0.0	0.8	0.0	0.0	0.0	0.0	0.1	0.0	0.0	0.1	0.1	98.6	722	M2	Right maxilla
0.0	0.0	0.3	0.0	0.0	0.0	0.0	1.3	0.0	0.3	0.0	0.0	0.1	0.8	0.0	0.0	0.8	96.1	713	M1	
0.0	0.0	0.2	0.0	0.0	0.2	0.3	1.4	0.0	1.4	0.8	0.3	5.7	17.6	1.4	1.9	2.4	66.6	637	P4	
0.0	0.0	0.2	0.0	0.0	0.0	0.7	2.4	0.0	2.4	0.0	0.0	0.4	0.4	2.0	3.6	3.6	84.2	450	P3	
0.0	0.0	0.0	0.0	0.3	0.0	0.3	1.5	0.0	5.1	0.0	0.0	0.0	0.0	4.1	3.6	6.4	78.7	389	P2	
0.0	0.0	0.0	0.0	0.3	0.0	0.8	1.9	0.5	3.2	0.0	0.0	0.0	0.0	0.8	5.0	1.6	86.0	378	P1	
0.0	0.0	0.0	0.6	0.0	0.0	1.5	2.4	0.3	0.9	0.0	0.0	2.7	2.1	0.9	1.2	0.3	87.1	333	C	
0.0	0.0	0.2	1.2	0.0	0.0	2.3	1.1	0.3	0.5	0.2	0.2	1.7	0.3	1.4	3.7	2.1	85.0	655	I3	
0.0	0.0	0.0	0.8	0.0	0.0	2.3	0.8	0.1	0.3	0.0	0.1	1.3	0.8	1.2	8.0	3.4	81.0	767	I2	
0.0	0.0	0.0	0.8	0.0	0.0	1.9	0.9	0.1	0.5	0.0	0.4	2.0	1.0	3.4	7.6	6.5	74.9	788	I1	
0.0	0.0	0.0	0.7	0.0	0.0	1.8	0.8	0.0	0.5	0.1	0.4	0.8	0.8	2.8	6.3	7.1	78.0	759	I1	Left maxilla
0.0	0.0	0.0	0.8	0.0	0.0	2.0	0.9	0.0	0.1	0.0	0.1	1.2	0.4	2.5	6.6	4.6	80.8	762	I2	
0.2	0.0	0.2	1.0	0.0	0.0	2.2	1.4	0.0	0.3	0.0	0.0	1.1	0.5	0.8	4.0	2.7	85.6	626	I3	
0.3	0.0	0.0	0.3	0.0	0.0	2.1	2.4	0.9	0.9	0.0	0.3	1.8	4.4	0.0	0.9	1.2	84.6	338	C	
0.0	0.0	0.0	0.0	0.3	0.0	1.6	1.9	0.5	2.4	0.0	0.3	0.0	0.0	1.6	3.7	3.5	84.2	374	P1	
0.0	0.0	0.0	0.0	0.0	0.0	1.2	1.4	0.0	4.6	0.0	0.0	0.2	0.2	5.3	4.4	8.6	74.0	431	P2	
0.0	0.0	0.0	0.0	0.0	0.0	0.9	2.0	0.0	2.6	0.0	0.0	0.0	0.2	1.5	5.3	3.5	83.9	453	P3	
0.2	0.0	0.0	0.0	0.2	0.2	0.3	1.5	0.2	1.8	1.5	0.2	4.7	17.7	1.3	2.1	3.3	65.0	615	P4	
0.1	0.0	0.3	0.0	0.0	0.0	0.1	1.1	0.0	0.3	0.0	0.0	0.1	0.1	0.1	0.1	1.4	96.2	736	M1	
0.0	0.0	0.4	0.0	0.0	0.0	0.0	0.6	0.0	0.1	0.0	0.0	0.0	0.1	0.0	0.0	0.3	98.2	775	M2	

C: canine; I: incisor; M: molar; P: premolar; RF: root fracture; CCRF: complicated crown-root fracture; CCF: complicated crown fracture; UCRF: uncomplicated crown-root fracture; UCF: uncomplicated crown fracture.

**Table 3:** Prevalence of oral diseases in each tooth among mandibular extracted teeth.

Prevalence of Oral Disease (%)																				
ONJ	MF	CA	MA	DC	DI	OT	CCUS	IP	TR	TF					FN	RRT	PD	Number of Extracted Teeth	Categories	
										UCF	UCRF	CCF	CCRF	RF						
0.0	0.0	0.0	0.0	0.0	0.0	0.4	0.8	1.5	1.1	0.0	0.0	0.0	0.0	0.0	0.0	0.0	96.2	265	M3	Right mandible
0.0	0.0	0.3	0.0	0.0	0.0	0.3	1.6	0.0	1.2	0.2	0.0	0.0	0.0	0.2	0.0	0.3	95.8	572	M2	
0.0	0.8	0.8	0.0	0.0	9.3	1.3	2.4	0.0	2.9	0.0	0.0	1.9	1.1	0.0	0.8	0.5	78.0	378	M1	
0.0	0.5	0.0	0.0	0.0	0.0	1.5	2.7	0.0	10.2	0.2	0.0	0.2	0.5	3.4	2.2	7.8	70.9	412	P4	
0.0	0.0	0.0	0.0	0.3	0.0	1.8	1.8	0.0	10.2	0.0	0.3	0.3	0.6	5.7	3.9	8.1	67.1	334	P3	
0.0	0.3	0.0	0.0	1.0	0.0	1.0	1.4	0.0	9.2	0.0	0.3	0.3	0.0	7.5	2.4	10.6	65.8	292	P2	
0.0	0.3	0.0	0.0	6.4	0.0	1.1	1.1	39.1	3.1	0.0	0.0	0.0	0.0	0.0	2.2	2.8	43.9	358	P1	
0.0	0.0	0.0	0.5	0.5	0.0	2.3	3.7	1.4	0.9	0.0	0.0	1.9	2.3	0.5	0.9	0.9	84.2	215	C	
0.0	0.0	0.0	0.3	0.0	0.0	2.0	1.0	0.2	0.0	0.0	0.0	0.8	0.0	3.1	2.0	2.6	88.0	608	I3	
0.0	0.0	0.0	0.0	0.0	0.0	1.5	1.1	0.2	0.0	0.0	0.2	0.6	0.0	4.3	1.4	3.7	87.0	648	I2	
0.0	0.0	0.0	0.1	0.0	0.0	1.9	0.9	0.6	0.1	0.0	0.0	0.3	0.4	4.6	1.3	6.1	83.7	700	I1	
0.0	0.0	0.0	0.1	0.0	0.0	1.1	0.8	0.6	0.1	0.0	0.1	0.1	0.1	6.0	1.5	5.6	83.7	719	I1	Left mandible
0.1	0.0	0.0	0.1	0.00	0.0	1.2	1.0	0.1	0.1	0.0	0.0	0.1	0.3	5.1	0.7	3.9	87.1	672	I2	
0.2	0.0	0.0	0.3	0.0	0.0	1.5	1.0	0.2	0.2	0.2	0.0	0.0	0.0	3.2	2.4	3.1	87.8	617	I3	
0.0	0.0	0.0	0.0	0.4	0.0	3.1	3.6	2.4	2.2	0.0	0.0	1.8	0.1	0.0	1.3	0.9	83.6	225	C	
0.0	0.0	0.0	0.0	8.2	0.0	1.4	1.6	38.5	1.9	0.0	0.0	0.0	0.0	0.0	2.2	1.6	44.5	366	P1	
0.0	0.0	0.0	0.0	2.8	0.0	0.7	1.8	0.4	8.8	0.0	0.0	0.4	0.0	9.5	1.8	9.5	64.6	285	P2	
0.0	0.0	0.0	0.0	0.0	0.0	0.3	2.8	0.0	9.6	0.0	0.0	0.9	0.9	5.3	2.5	8.7	68.9	322	P3	
0.0	0.5	0.0	0.0	0.0	0.2	0.7	2.5	0.0	9.2	0.0	0.0	0.2	0.2	3.9	2.5	9.7	70.3	435	P4	
0.3	2.1	0.3	0.0	0.0	8.8	0.8	2.6	0.0	2.6	0.0	0.0	1.8	1.0	0.3	0.3	0.8	78.6	388	M1	
0.0	0.0	0.4	0.0	0.0	0.2	0.4	1.6	0.0	1.4	0.0	0.0	0.0	0.0	0.4	0.0	0.5	95.1	552	M2	
0.0	0.0	0.0	0.0	0.0	0.0	0.4	1.9	1.1	1.5	0.0	0.0	0.4	0.0	0.4	0.0	0.8	93.5	262	M3	

C: canine; I: incisor; M: molar; P: premolar; RF: root fracture; CCRF: complicated crown-root fracture; CCF: complicated crown fracture; UCRF: uncomplicated crown-root fracture; UCF: uncomplicated crown fracture.

## Data Availability

The data presented in this study are available on request from the corresponding author.
